# Supplementary material for: Feature ranking and network analysis of global financial indices
Source: PLoS One. 2022 Jun 3;17(6):e0269483. doi: 10.1371/journal.pone.0269483 (PMC9165829; doi:10.1371/journal.pone.0269483)
Supplement: S1 Appendix — (DOCX) [file pone.0269483.s001.docx]

| **Abbreviation** | **Full Name** |
| --- | --- |
| AUS | Australia |
| MAL | Malaysia |
| HK | Hong Kong |
| TWN | Taiwan |
| CHI | China |
| JAP | Japan |
| SING | Singapore |
| KOR | Korea |
| IND | India |
| USA | United States of America |
| CAN | Canada |
| UK | United Kingdom |
| GER | Germany |
| FRA | France |
| SWI | Switzerland |
| BRA | Brazil |
| MEX | Mexico |
| ARG | Argentina |
| ISR | Israel |
| SPN | Spain |
| EGY | Egypt |

**Stock markets observed in this research**

**Table 1. Details of 21 stock markets monitored in this research.**
