# Supplementary material for: Feature ranking and network analysis of global financial indices
Source: PLoS One. 2022 Jun 3;17(6):e0269483. doi: 10.1371/journal.pone.0269483 (PMC9165829; doi:10.1371/journal.pone.0269483)
Supplement: Attachment — Submitted filename: Response to Reviewers.docx [file pone.0269483.s002.docx]

**Rebuttal letter**

PONE-D-22-06124

Feature ranking and network analysis of global financial indices

PLOS ONE

27 April 2022

We thank the editor and the reviewer for their valuable comments on our manuscript. Below is our response to each point raised by the academic editor and reviewer. We hope that we satisfyingly addressed them and that the manuscript will be now suited for publication.

Sincerely,

On behalf of all authors,

Dr. Ashadun Nobi

**Responds to Academic Editor**

Comment 1: Please ensure that your manuscript meets PLOS ONE's style requirements, including those for file naming.

***Response:*** File naming was edited to comply with the style requirements. We hopefully have no divergences from the style requirements now.

Comment 2: We suggest you thoroughly copyedit your manuscript for language usage, spelling, and grammar. If you do not know anyone who can help you do this, you may wish to consider employing a professional scientific editing service.

***Response:*** Thank you for your suggestion. We used “Grammarly” online software and “QuillBot” word extension to edit the spelling, grammar, and language usage. We edit our manuscript according to the requisitions of the editor and reviewer. The modifications are highlighted in bright green color, new insertions are highlighted in yellow, and deletions are highlighted in red in the “Revised Manuscript with Track Changes” file. We corrected spelling and grammatical mistakes and enhanced some language usage without changing the meaning of the sentences which are not highlighted. Regarding the use of professional editing, we don’t afford to pay the required payment.

Comment 3: Please note that PLOS ONE has specific guidelines on code sharing for submissions in which author-generated code underpins the findings in the manuscript. In these cases, all author-generated code must be made available without restrictions upon publication of the work. Please review our guidelines at https://journals.plos.org/plosone/s/materials-and-software-sharing#loc-sharing-code and ensure that your code is shared in a way that follows best practice and facilitates reproducibility and reuse.

***Response:*** The python 3.7 implemented codes related to this research work are made publicly available on Github and can be found at https://github.com/Rakib-Mahmud/Feature-Ranking-and-Network-Analysis. This repository provides a generalized version of the codes, which are not implemented solely for this research.

Comment 4: We note that the grant information you provided in the ‘Funding Information’ and ‘Financial Disclosure’ sections do not match.

When you resubmit, please ensure that you provide the correct grant numbers for the awards you received for your study in the ‘Funding Information’ section.

***Response:*** We will correct the mismatches with the below information when we resubmit. Please do this for us in case we don’t find this option in the online submission system while resubmitting. Thank you.

Funded Author: Mahmudul Islam Rakib

Grand Number: 20FS34427

Funder Name: ICT Division of Bangladesh

Funder Web-address: http://ims.ictd.gov.bd/

Funder’s Role in the research: The funders had no role in study design, data collection and analysis, decision to publish, or preparation of the manuscript.

Comment 5: Thank you for stating the following in the Acknowledgments Section of your manuscript:

[This work was supported by the ICT Division of Bangladesh under grant number 20FS34427. The author thanks Prof. Jae Woo Lee for his valuable comments.]

We note that you have provided funding information that is not currently declared in your Funding Statement. However, funding information should not appear in the Acknowledgments section or other areas of your manuscript. We will only publish funding information present in the Funding Statement section of the online submission form.

Please remove any funding-related text from the manuscript and let us know how you would like to update your Funding Statement. Currently, your Funding Statement reads as follows:

[NO. The funders had no role in study design, data collection and analysis, decision to

publish, or preparation of the manuscript.]

Please include your amended statements within your cover letter; we will change the online submission form on your behalf.

***Response:*** We removed the funding-related text from the Acknowledgments Section of our manuscript. Please update the Funding Statement as [This work was supported by the ICT Division of Bangladesh under grant number 20FS34427. The funders had no role in study design, data collection and analysis, decision to publish, or preparation of the manuscript.]

Comment 6: We note that you have indicated that data from this study are available upon request. PLOS only allows data to be available upon request if there are legal or ethical restrictions on sharing data publicly. For more information on unacceptable data access restrictions, please see http://journals.plos.org/plosone/s/data-availability#loc-unacceptable-data-access-restrictions.

***Response:*** The data underlying the results presented in the study are available from Yahoo Finance (https://finance.yahoo.com/world-indices).

Comment 7: Please review your reference list to ensure that it is complete and correct. If you have cited papers that have been retracted, please include the rationale for doing so in the manuscript text, or remove these references and replace them with relevant current references. Any changes to the reference list should be mentioned in the rebuttal letter that accompanies your revised manuscript. If you need to cite a retracted article, indicate the article’s retracted status in the References list and also include a citation and full reference for the retraction notice.

***Response:*** There is no change in the reference list.

**Responds to Reviewer #1**

Comment 1: In Eq. 6 you used the average value of elements of feature ranking matrix. Do you have a reason to choose the threshold as the average? Is it a rule of sum? You can give some comments when you change the threshold.

***Response:*** We applied static threshold that remains fixed every year and avoided dynamic threshold as it is assigned with high values during crises, so the finer network structure and topological properties are not seen. While selecting a static threshold, we found that the sum of each row of the feature ranking matrix is always one as the total probability of influence of features on the target is one, so the mean of the matrix for each year is unchangeable. Selecting the mean of elements of the feature ranking matrix as the threshold gave fine network properties through which we can identify crises and distribution of influence properly.

The equation follows a rule of product, i.e., the sum is performed $N*N$ times instead of $N+N$ times.

The network structure is sensitive to the threshold. So, when we alter the threshold, the topological properties of the financial network have changed. The topological properties of the network around the mean threshold are finer than other thresholds for understanding the market movement.

Comment 2: When you consider 2008, 2011, 2016 crisis, you use different length of the time series. For example, when you consider 2008 crisis, you use the previous time series before year 2008. Could you give the length of the time series for the training set? Is it the one-year time window to get the feature ranking matrix?

***Response:*** The length of the time series for the dataset is 3838 days. Then this dataset is divided into 15 segments considering 15 years using the one-year time window. In each time window, there are around 256 days.

Comment 3: In your definition, the self-influence F_ii is determined by the current return influenced by the previous return itself. Could you explain that the self-influence is small compared to other indices?

***Response:*** This study performed multivariate analyses, and the total influence on the target is distributed among multiple features, including the target index. Here, influence interprets the probability of contribution in predicting the target. From the time series, we observe that the returns of any index can change at random or follow some pattern. In the first situation, predicting the current returns from randomly changing previous returns of its own may score a lower contribution in predicting itself. But the time series of that index can be more related to the time series of other indices. In that case, self-influence is small compared to other indices. Conversely, when returns of an index follow some pattern, self-influence is high. Such an example is shown for Japan in Fig. 1b during the ESD crisis in 2011.

Comment 4: In Fig. 1b, you consider ESD crisis. Then, many Asian countries influenced by American and European countries. Could you explain why Asian countries are so sensitive to other markets.

***Response:*** In this analysis, we found that Asian stock markets are influenced largely by other markets, but in return, their influence on others is less. This means the states of the Asian markets are dependent on the other markets, especially during crises. From our observation, we presume that the Asian stock markets are not as developed as American and European; perhaps they are emerging and hence sensitive to other markets. We need further analysis in this regard, and we leave it for future investigation.

Comment 5: When you define the total influence in Eq. 8, it seems that the F_ij is unnormalized because F_tot (j) varies in a wide range. The absolute value of each F_ij has some meanings?

***Response:*** The absolute value of $F_{ij}$ means the influence of index $j$ on index $i$. Here, we analyzed the total impact of each index on others every year. For this, we used unnormalized $F_{ij}$ to retain its original value.

Comment 6: Is there any reason that many Asian countries in Fig. 3 don’t have in-degree?

***Response:*** In Fig. 3, the in-degree of a node indicates the number of influenced nodes by that node in the network. In this figure, some Asian countries like Taiwan do not have any in-degree, which means those countries have no substantial influence on any other country in the network. This is because each feature rank of those countries in the feature ranking matrix is smaller than the mean threshold during 2008.

Comment 7: In line 264 below Eq (9), “the adjacency matrix of the feature ranking network….” have to change as “the reconstructed adjacency matrix of the feature ranking network ….”.

***Response:*** We have changed it as you suggested.

Comment 8: In Eq. 9, I_N (j) is the number of out-degree for the node j because hat{A}_ij indicate that j  i(target) as hat{A}_ij >= theta.

***Response:*** In Eq. 9, $I_{N}(j)$ is the number of in-degree for the node $j$ because $\hat{A}_{ij}$ indicates that $i(target)$→ $j$, as $F_{ij}>\theta$. Here, $\hat{A}$ is the reconstructed adjacency matrix generated by comparing the elements of the feature ranking matrix $F$ with the threshold $\theta$. Note that the direction here shows dependency, i.e., target $i$ is dependent on or influenced by feature $j$.

Comment 9: Do you neglect the direction when you define the network density, average shortest path?

***Response:*** We do not neglect the direction while defining network density, average shortest path. Here, bi-direction is possible; hence the maximum number of possible links is $N(N-1)$, which is similar to the undirected network.

Comment 10: In Eq.12, you defined the local reaching centrality. Then it means that all possible nodes you follow out-going links starting from node i. For example, the node ijkl, then the node l is reachable from i to l. Do you mean this type of reaching? Could you explain clearly?

***Response:*** The local reaching centrality of node $i$ means the proportion of nodes in the network reachable from that node directly or via multiple hops. For instance, take a line network $i\to j\to k\to l$. Here, all the three nodes are reachable from node $i$ directly or via multi-hops. So its local reaching centrality is maximum. But two nodes are reachable from $j$, so its centrality is smaller than that of $i$. This way, no nodes are reachable from $l$, hence its local reaching centrality is minimum.

Comment 11: Check typos.

***Response:*** We have checked.
